# Minimally Invasive Coronary Artery Bypass Grafting for Predominantly Multivessel Coronary Artery Disease: Early Perioperative Outcomes of 149 Patients

**DOI:** 10.7759/cureus.116528

**Published:** 2026-09-19

**Authors:** Dryasir Khan, Ali Akbar Zehri, Rana Altaf

**Affiliations:** 1 Cardiac Surgery, Mukhtar A Sheikh Memorial Trust Hospital, Multan, PAK; 2 Cardiac Surgery, Sardar Fateh Muhammad Khan Buzdar Institute of Cardiology, Dera Ghazi Khan, PAK; 3 Surgery, The Aga Khan Hospital, Dar es Salaam, TZA; 4 Anesthesia and Critical Care, Mukhtar A Sheikh Memorial Trust Hospital, Multan, PAK

**Keywords:** cardiopulmonary bypass, coronary artery bypass, cross-clamp, mics-cabg, minimally invasive cardiac surgery, minimally invasive coronary artery bypass grafting, multivessel coronary artery disease, opcab, perioperative outcomes

## Abstract

Background

Minimally invasive coronary artery bypass grafting (MICS-CABG) has evolved from single-vessel revascularization to multivessel surgery without median sternotomy. Although favorable outcomes have been reported, experience remains concentrated in specialized centers. This study evaluated the demographic, operative, and early postoperative characteristics of patients undergoing MICS-CABG, off-pump coronary artery bypass grafting (OP-CABG) at a single institution.

Methods

This retrospective observational study included 149 CABG/OPCAB procedures identified from an institutional database of 186 cardiac surgical records. Demographics, cardiovascular risk factors, operative details, number of grafts, cardiopulmonary bypass (CPB) and cross-clamp times, postoperative ventilation, chest drainage, transfusion requirements, and documented complications were analyzed. Continuous variables were summarized as mean ± standard deviation and median with interquartile range (IQR), and categorical variables as frequencies and percentages. Missing data were not imputed.

Results

The mean age was 57.8 ± 9.5 years; 139 (93.3%) patients were men. Diabetes mellitus was present in 86 patients (57.7%), hypertension in 100 (67.1%), and tobacco exposure in 65 (43.6%). Procedures included eight one-graft, 41 two-graft, 86 three-graft, and 13 four-graft operations; one procedure lacked standardized graft documentation. Overall, 140/149 (94.0%) procedures had two or more explicitly coded grafts.

CPB data were available for 137 patients, with a mean duration of 170.2 ± 37.4 minutes and a median of 168 minutes (IQR: 148-190). Mean cross-clamp time was 94.6 ± 30.5 minutes. Ventilation data were available for 146 patients, with a mean duration of 7.6 ± 4.4 hours and a median of six hours (IQR: 4-11). Chest drainage was documented in 134 patients, with a mean of 899 ± 510 mL. Early postoperative outcomes included conversion to sternotomy in nine patients (6.0%), return to the operating room for bleeding in nine (6.0%), stroke in 3 (2.0%), respiratory insufficiency in two (1.3%), new-onset atrial fibrillation in 36 (24.2%), and 30-day or in-hospital mortality in three (2.0%). Outcome-specific denominators were based on the number of patients for whom the corresponding variable was available.

Conclusion

This institutional experience demonstrates the feasibility of minimally invasive coronary surgery for predominantly multivessel revascularization. Most procedures involved two or more grafts, with three-graft surgery being the most common. Postoperative ventilation was generally short; however, incomplete complication and transfusion documentation limits interpretation of other perioperative outcomes. Prospective standardized data collection and comparative studies with conventional CABG are warranted.

## Introduction

Coronary artery disease (CAD) remains one of the leading causes of cardiovascular morbidity and mortality worldwide. Coronary revascularization with either percutaneous coronary intervention (PCI) or coronary artery bypass grafting (CABG) remains an important component of treatment for patients with significant obstructive CAD, with the optimal strategy determined by coronary anatomy, clinical presentation, comorbidities, ventricular function, anticipated completeness of revascularization, and patient preferences [1-4].

CABG has traditionally been performed through median sternotomy. Although median sternotomy provides excellent exposure and remains the standard surgical approach for complex coronary revascularization, it is associated with sternal trauma, postoperative activity restrictions, sternal wound complications, and recovery-related morbidity [5,6]. These considerations have encouraged the development of less invasive approaches that attempt to preserve the effectiveness of surgical revascularization while reducing the consequences of sternotomy.

Minimally invasive coronary artery bypass grafting (MICS-CABG) encompasses a spectrum of procedures performed through limited thoracic access, generally through a left anterior or anterolateral minithoracotomy, with or without cardiopulmonary bypass. Unlike minimally invasive direct coronary artery bypass (MIDCAB), which traditionally focuses on left internal thoracic artery (LITA) revascularization of the left anterior descending artery (LAD), MICS-CABG can be used to achieve multivessel coronary revascularization [7,8].

The concept of multivessel minimally invasive coronary surgery was introduced more than two decades ago, with early reports demonstrating the feasibility of performing multiple coronary anastomoses through limited access [9,10]. Early clinical experience subsequently demonstrated that minimally invasive techniques could be extended from single-vessel procedures to multivessel coronary revascularization [11]. Subsequent technical development has allowed MICS-CABG to evolve into an approach capable of two-, three-, and four-vessel coronary revascularization in selected patients.

In a systematic review of 26 studies involving 7,556 patients undergoing MICS-CABG for multivessel CAD, Sef et al. reported an average patient age of 63.3 years and an average of approximately 2.8 grafts per patient. Early mortality and stroke averaged 0.6% and 0.4%, respectively [1]. These findings suggest that, in experienced centers, minimally invasive access can be extended beyond isolated LAD disease without necessarily compromising early safety.

Long-term data have also become increasingly available. Guo et al. reported 566 patients undergoing MICS-CABG over a 17-year period, with 283 patients undergoing multivessel grafting. At 12 years, overall survival was 82.2%, supporting the durability of minimally invasive surgical revascularization in appropriately selected patients [2].

Similarly, Rufa et al. compared minimally invasive multivessel CABG with MIDCAB in more than 1,000 patients and reported comparable long-term freedom from angina, myocardial infarction, and repeat revascularization [3]. Their findings support the concept that adding grafts to the minimally invasive approach does not necessarily result in inferior long-term clinical outcomes.

Robotic coronary revascularization represents another minimally invasive strategy, with its implementation dependent on appropriate patient selection, specialized technology, team experience, and structured program development [12]. Wider adoption of MICS-CABG depends not only on appropriate patient selection and institutional expertise but also on structured surgeon and team training. Because minimally invasive access restricts exposure and requires adaptation of conventional coronary techniques, training should build on proficiency in off-pump coronary artery bypass grafting (OPCAB) and incorporate stepwise technical development, careful case selection, mentorship, and progressive procedural complexity. Sarfaraz et al. proposed a structured framework emphasizing these principles to facilitate safe and well-tolerated adoption of minimally invasive coronary surgery [13]. Importantly, training should be considered competency-based rather than defined by a fixed duration, as proficiency depends on prior coronary surgical experience, procedural volume, mentorship, and institutional support. Robotic CABG literature similarly identifies technical learning curves, procedural complexity, and institutional resources as important determinants of widespread adoption [12,14,15].

Minimally invasive coronary surgery may also reduce some of the morbidity associated with sternotomy. Total arterial multivessel MICS-CABG has been particularly attractive because avoiding median sternotomy may permit use of bilateral internal thoracic arteries without exposing the sternum to the same wound-related risks associated with bilateral internal thoracic artery harvest through sternotomy [16].

Nevertheless, most published MICS-CABG experience originates from high-volume specialist centers. Evidence from developing minimally invasive programs and from regions where this technology is less widely established remains comparatively limited [11,17]. Real-world institutional series are therefore valuable for defining the patient profile, operative complexity, and early postoperative recovery achievable during program development.

The present study was undertaken to describe the early perioperative outcomes of 149 consecutive patients undergoing minimally invasive CABG or OPCAB at a single institution. The specific objectives were to characterize patient demographics and cardiovascular risk factors, determine the distribution of graft numbers, quantify CPB and cross-clamp duration, evaluate postoperative ventilation and chest drainage, describe blood-product utilization, and report documented early postoperative complications.

The study was reported according to the principles of the Strengthening the Reporting of Observational Studies in Epidemiology (STROBE) statement [18].

## Materials and methods

This was a retrospective observational cohort study based on a prospectively maintained institutional cardiac surgery database. All procedures were performed by a single consultant cardiac surgeon with established experience in conventional coronary artery bypass surgery. The study period encompassed the development and maturation of the institution's minimally invasive coronary revascularization program; therefore, the cohort included cases performed during the surgeon and surgical team's learning curve. Procedural complexity was increased progressively with accumulating experience and appropriate patient selection. The study period represented the initial institutional experience with MICS-CABG, and the operating surgeon had not performed an independent series of MICS-CABG procedures at this institution before January 2023.

The original database contained 186 cardiac surgical records. Of these, 149 patients underwent minimally invasive coronary revascularization procedures, coded as MICS-CABG or MIDCAB, between January 2023 and December 2025 and fulfilled the study eligibility criteria. The remaining records predominantly represented isolated valvular surgery, congenital cardiac procedures, and other minimally invasive cardiac procedures and were not included in the study cohort. The inclusion and exclusion criteria are presented in Table 1.

**Table 1:** Inclusion and exclusion criteria MICS-CABG: Minimally invasive coronary artery bypass grafting; MIDCAB: minimally invasive direct coronary artery bypass; CABG: coronary artery bypass grafting

Inclusion criteria	Exclusion criteria
Elective isolated minimally invasive coronary artery bypass grafting (MICS-CABG or MIDCAB) performed between January 2023 and December 2025	Emergency procedures
Age 30–80 years	Valvular heart disease
Male or female sex	Combined procedures, such as CABG with aortic valve replacement (AVR) or mitral valve replacement (MVR)
Preoperative left ventricular ejection fraction (LVEF) >35%	Congenital heart disease
Complete coronary revascularization achieved	Poor distal runoff or unsuitable distal coronary target vessel precluding satisfactory surgical revascularization
	Severe chest deformity
	Severe chronic obstructive pulmonary disease (COPD)
	Stroke within four weeks before surgery
	Acute myocardial infarction within seven days before surgery
	Left ventricular ejection fraction (LVEF) <35%
	Cardiomegaly, defined as left ventricular end-diastolic diameter >6.0 cm

The following variables were extracted from the database: age, sex, body weight, body surface area, diabetes mellitus, hypertension, tobacco exposure, family history, serum creatinine, surgical procedure, number of grafts, CPB duration, aortic cross-clamp duration, graft conduit information, duration of mechanical ventilation, chest drainage, blood-product utilization, and documented postoperative complications. Patients were positioned with the left chest elevated 30 to 40 degrees to widen the intercostal space (ICS). All procedures were done using conventional ventilation, through an 8-10 cm left mini-thoracotomy (with 1/3 of the incision medial to the mid-clavicular line) in the fourth or fifth ICS to expose the left internal thoracic artery. The LITA was harvested along its full length under direct vision. The saphenous vein graft was harvested using the non-touch technique. Patients with multi-vessel disease were operated using cardiopulmonary bypass through femoral arterial and venous cannulation and cardioplegic arrest using Del Nido cardioplegia, while those with single-vessel disease were done on a beating heart. Two deep pericardial stitches were routinely fixed in order to expose the lateral and inferior walls of the heart. The principal outcomes were early perioperative measures: number of coronary grafts; CPB duration; aortic cross-clamp duration; postoperative mechanical ventilation duration; chest drainage; blood-product utilization; and documented postoperative complications. Postoperatively, patients are given a stat dose of enteric-coated acetylsalicylic acid 325 mg within six hours of procedure. All patients are treated with medical therapy as with conventional CABG via sternotomy, including aspirin, β-blockers, and anticholesterol agents. Patients undergoing MICS-CABG with a radial artery graft are prescribed calcium channel blockers for one year. All patients were followed for at least 30 days after surgery. Operative mortality was defined as death occurring within 30 days of surgery, regardless of location, or during the index hospitalization, irrespective of the duration of hospitalization. Thirty-day follow-up was complete for all patients. Stroke was defined as a permanent, new, focal neurologic deficit occurring either intraoperatively or postoperatively. Respiratory insufficiency was defined as a cumulative requirement for intubation and ventilation of 72 hours or more at any time during the postoperative stay. New-onset renal failure was defined as the need for temporary or permanent renal dialysis of any type. Transfusion rates were determined by the proportion of patients who received at least one transfusion of any blood product. Continuous variables were summarized as mean ± standard deviation (SD) and median with interquartile range (IQR). Categorical variables were expressed as numbers and percentages. For each variable, the denominator was based on the number of available observations.

## Results

Patient characteristics

The study cohort comprised 149 patients. The mean age was 57.8 ± 9.5 years, with a median age of 58 years (IQR: 52-65). The youngest patient was 30 years old, and the oldest was 80 years old.

There were 139 male patients (93.3%) and 10 female patients (6.7%). Mean body weight was 76.9 ± 12.9 kg, while mean body surface area was 1.88 ± 0.18 m². Serum creatinine was available for 148 patients and had a mean value of 1.01 ± 0.29 mg/dL. The preoperative characteristics and cardiovascular risk factors are shown in Table 2.

**Table 2:** Preoperative characteristics and cardiovascular risk factors

Variable	Value
Total number of patients	149
Age, mean ± standard deviation	57.8 ± 9.5 years
Age, median (IQR)	58 (52-65) years
Age range	30-80 years
Male sex	139 (93.3%)
Female sex	10 (6.7%)
Weight, mean ± standard deviation	76.9 ± 12.9 kg
BSA, mean ± standard deviation	1.88 ± 0.18 m²
Creatinine, mean ± standard deviation	1.01 ± 0.29 mg/dL
Diabetes mellitus	86 (57.7%)
Hypertension	100 (67.1%)
Tobacco exposure	65 (43.6%)
Positive family history	76 (51.0%)
Negative family history	73 (47.7%)
Ejection fraction %	42.4 ± 11.5
Triple-vessel disease %	99 (66.4%)

The relatively young age of the cohort compared with many published MICS-CABG series may reflect local patient selection and the institutional referral population. In the systematic review by Sef et al., the average age across published MICS-CABG cohorts was 63.3 years [1]. The marked male predominance in the present cohort is consistent with the sex distribution observed in many contemporary surgical coronary revascularization series [1,3,5].

Cardiovascular risk factors

Diabetes mellitus was present in 86 patients (57.7%), while 63 patients (42.3%) were documented as not having diabetes. Hypertension was present in 100 patients (67.1%), whereas 49 patients (32.9%) did not have hypertension. After standardization of the smoking field, tobacco exposure was documented in 65 patients (43.6%). Family history was positive in 76 patients (51.0%), negative in 71 patients (47.7%), and indeterminate in two patients (1.3%).

The high prevalence of diabetes and hypertension demonstrates that the MICS program was not limited to patients without significant cardiovascular comorbidity. This is clinically relevant because diabetes mellitus and other cardiovascular risk factors frequently coexist with extensive coronary disease and may influence the choice between surgical and percutaneous revascularization [6,7]. The contemporary MICS-CABG literature similarly includes patients with substantial cardiovascular risk-factor burdens, supporting the expanding role of minimally invasive surgery beyond isolated low-risk coronary lesions [1,2].

Distribution of coronary grafts

The operation field demonstrated the following graft distribution: eight one-graft procedures (5.4%), 41 two-graft procedures (27.5%), 86 three-graft procedures (57.7%), and 13 four-graft procedures (8.7%). One hundred forty of these 149 patients (94.0% of the total cohort) had two or more grafts.

The operative characteristics and postoperative results are shown in Table 3. 

**Table 3:** Operative characteristics and postoperative results (N=149) LITA: Left internal thoracic artery; CPB: cardiopulmonary bypass

No. of grafts, n	2.1±0.7 (range: 1–4)
1 graft	8 (5.4%%)
2 grafts	42 (28.18%)
3 grafts	86 (57.7%)
4 grafts	13 (8.7%)
Use of CPB, n	141 (94.6%)
CPB time (min)	170.2 ± 37.4 min
Cross-clamp time (min)	94.6 ± 30.5 min
Ventilation time (hrs)	7.6 ± 4.4 hours
Conversion to sternotomy, n	9 /149(6%)
Bleeding	5
Pulmonary artery injury	2
LITA injury	2
Return to operating room, n	9 /149(6%)
Bleeding	9
Perioperative transfusion, n	61/149 (40.1%)
No. of units	3.2±3.4
Chest drainage (ml)	899 ± 510 mL
Stroke, n	3 (2.01%)
Respiratory insufficiency, n	2 /149(1.34%)
New-onset atrial fibrillation, n	36 /149(24.4%)
Length of stay, days	5.9±3.4 (range: 1–86)
Death, n	3 /149(2.01%)

Three-graft surgery represented the largest operative category, accounting for nearly 58% of the cohort. This is an important feature of the present series because it demonstrates that the institutional MICS program was predominantly being used for multivessel coronary revascularization, rather than being restricted to LITA-to-LAD surgery.

The findings are consistent with the contemporary literature. Sef et al. reported an average of 2.8 grafts per patient in their systematic review of 26 MICS-CABG studies involving 7,556 patients [1]. Verevkin et al. also reported favorable five-year outcomes after total arterial multivessel MICS-CABG, reinforcing the feasibility of performing extensive coronary revascularization through minimally invasive access [5].

The cohort included both on pump CABG and off pump CAB procedures. Seven patients underwent OPCAB (single graft left internal thoracic artery to LAD) (MICS). The remaining patients underwent CABG procedures, including two-, three-, and four-graft operations.

The presence of both on-pump and off-pump surgery illustrates that MICS is primarily an access strategy rather than a single perfusion strategy. Contemporary minimally invasive coronary surgery may be performed with CPB, without CPB, or through hybrid strategies depending on anatomy, surgeon experience, patient characteristics, and institutional preference [1,3,4]. Table 3 illustrates the operative and postoperative results.

Cardiopulmonary bypass and cross-clamp times

The mean CPB duration was 170.2 ± 37.4 minutes, with a median of 168 minutes (IQR: 148-190 minutes). The observed range was 69-274 minutes. The mean cross-clamp duration was 94.6 ± 30.5 minutes, with a median of 93 minutes (IQR: 74-110 minutes) and a range of 40-221 minutes. The relatively prolonged CPB duration should be interpreted in the context of the complexity of the cohort, in which three-graft procedures were predominant. Furthermore, not all patients underwent CPB, meaning that the reported CPB values represent only those procedures for which CPB duration was recorded. MICS-CABG can be performed either on-pump or off-pump, and published series differ substantially in their use of CPB [3,4].

The median ventilation duration of six hours indicates that early postoperative extubation was achievable in the majority of patients. Early extubation and accelerated recovery are among the proposed benefits of minimally invasive cardiac surgery, although these outcomes are influenced by anesthesia protocols, ICU practices, patient selection, operative complexity, and institutional pathways [8,9]. The present study cannot determine whether the observed ventilation duration is superior to conventional sternotomy CABG because no control group was available.

The mean drainage volume was 899 ± 510 mL, with a median of 775 mL (IQR, 592.5-1090 mL). The range was 110-3320 mL.

## Discussion

The present study describes the early perioperative profile of 149 patients undergoing minimally invasive coronary revascularization. The principal finding is that the institutional MICS program was predominantly used for multivessel coronary surgery. Among the 149 procedures, 140 had clearly identifiable two-, three-, or four-graft configurations, representing 94.0% of the total cohort.

Three-graft surgery was the dominant operative category, accounting for 86 patients. These findings are important because the clinical value of MICS-CABG increasingly depends on its ability to reproduce the completeness of revascularization associated with conventional CABG while preserving the potential recovery benefits of avoiding sternotomy [1,3,5].

Historically, minimally invasive coronary surgery was primarily associated with MIDCAB and LITA-to-LAD grafting. The development of multivessel MICS-CABG represents an important extension of this concept. The contemporary systematic review by Sef et al. identified 7,556 patients across 26 studies and demonstrated that MICS-CABG could be performed for multivessel CAD with an average of 2.8 grafts per patient [1].

The current cohort had a similarly substantial graft burden, with three-graft procedures representing the majority. This suggests that the institutional program has moved beyond a single-vessel minimally invasive model toward a broader multivessel revascularization strategy.

The ability to achieve multivessel revascularization through limited thoracic access is clinically relevant because completeness of revascularization remains an important determinant of outcomes following CABG [6,7].

The present cohort shares several characteristics with contemporary published MICS-CABG populations. The mean age was 57.8 years, compared with 63.3 years in the systematic review by Sef et al. [1]. The proportion of men was 93.3%, compared with an average of 77.8% across the studies included in the systematic review [1].

The current cohort also demonstrated a high prevalence of diabetes and hypertension, emphasizing that minimally invasive surgery is being applied to patients with substantial cardiovascular risk profiles. Guo et al. reported long-term outcomes in 566 MICS-CABG patients, including 283 with multivessel grafting. Their 12-year survival was 82.2% [2]. Although the current study lacks long-term follow-up, the operative profile of our cohort supports the feasibility of the same general surgical concept.

Three-graft procedures accounted for 57.7% of the current cohort. This is particularly important because the ability to provide multiple grafts is one of the principal differences between MICS-CABG and traditional MIDCAB. Rufa et al. studied more than 1,000 patients undergoing minimally invasive coronary surgery and reported comparable long-term outcomes between MICS-CABG and MIDCAB [3]. Their study included patients receiving two and three grafts and demonstrated that multivessel minimally invasive surgery could achieve durable outcomes comparable with single-vessel minimally invasive surgery [3].

Verevkin et al. reported five-year outcomes after total arterial multivessel MICS-CABG and highlighted the potential advantage of combining minimally invasive access with arterial grafting [5]. These studies collectively support the concept that minimally invasive access does not necessarily require compromise in the extent of surgical revascularization.

The mean CPB time in the present study was 170 minutes. However, this finding must be interpreted cautiously. MICS-CABG is not synonymous with off-pump CABG. Depending on surgical strategy, CPB can be avoided or employed through minimally invasive cannulation techniques [1,3,4].

The current dataset included seven OPCAB procedures; thus, the mean CPB time primarily reflects the subgroup in whom CPB was used and recorded. The heterogeneity of CPB strategies in contemporary MICS-CABG literature makes direct comparison of absolute CPB times between institutions problematic.

The median postoperative ventilation duration was six hours. Early extubation is an important component of enhanced recovery following cardiac surgery. Minimally invasive access may facilitate early mobilization by reducing sternal pain and eliminating restrictions related to sternotomy [8,9].

However, postoperative ventilation is multifactorial and is influenced by age, pulmonary disease, operative duration, transfusion, bleeding, CPB duration, anesthetic practice, temperature management, and ICU protocols. Therefore, the present ventilation results should be considered descriptive. Nevertheless, the fact that the majority of patients were extubated within the first postoperative day supports the feasibility of an accelerated recovery pathway.

The median chest drainage was 775 mL. Although this provides useful descriptive information, the absence of a standardized drainage interval prevents interpretation as a specific 12- or 24-hour blood-loss measurement. Bleeding is an important consideration in MICS because limited access can make exposure and re-exploration more technically challenging.

Rodriguez et al. reported a 2% reoperation rate for bleeding in their 306-patient experience [4]. The current dataset did not permit reliable estimation of reoperation for bleeding. The heterogeneous transfusion documentation is another important limitation.

Contemporary cardiac-surgery guidelines emphasize standardized perioperative blood-management strategies because transfusion is associated with adverse outcomes and should be minimized when clinically appropriate [16]. The 2024 EACTS (European Association for Cardio-Thoracic Surgery) guidelines on perioperative medication and contemporary patient-blood-management literature provide a framework for improving standardized perioperative management in adult cardiac surgery [16].

Contemporary MICS-CABG studies generally report specific outcomes including operative mortality, stroke, myocardial infarction, atrial fibrillation, reoperation, conversion to sternotomy, renal failure, pulmonary complications, and wound complications. Sef et al. reported an average early mortality of 0.6% and stroke of 0.4% in their systematic review [1]. Rodriguez et al. reported a 3.3% sternotomy conversion rate and 2% reoperation for bleeding in their 306-patient cohort [4]. These outcomes are comparable with our data.

The learning curve is one of the most important challenges associated with MICS-CABG. Rodriguez et al. demonstrated that sternotomy conversion decreased from 5.2% during the earlier portion of their experience to 1.3% during the later period [4]. Contemporary robotic CABG evidence similarly identifies learning curve, institutional volume, procedural complexity, and team experience as important determinants of outcomes [12,14,19].

Hwang et al., in a systematic review and meta-analysis encompassing more than 6,000 robotic MIDCAB and 1,700 TECAB patients, reported an overall conversion-to-sternotomy rate below 3.2%, while long-term graft patency was approximately 96% in both robotic MIDCAB and TECAB (totally endoscopic coronary artery bypass) cohorts [8]. Although robotic CABG differs technically from conventional MICS-CABG, these findings reinforce the importance of experience and standardized pathways.

The most important unanswered question is whether MICS-CABG provides equivalent coronary revascularization and survival with fewer perioperative complications compared with conventional sternotomy CABG. Ong et al. performed a propensity-matched analysis comparing MICS-CABG with conventional CABG and found that minimally invasive surgery was associated with shorter postoperative hospitalization in selected groups, although procedural duration was longer in some multivessel patients [19]. Gong et al. similarly reported comparable completeness of revascularization and intermediate-term cardiovascular outcomes between MICS-CABG and conventional CABG after propensity matching [15].

These observations are consistent with the concept that MICS-CABG can provide the fundamental objective of CABG-complete coronary revascularization-while potentially improving recovery. However, the existing evidence remains predominantly observational.

The 2021 American College of Cardiology (ACC), the American Heart Association (AHA), and the Society for Cardiovascular Angiography and Interventions (SCAI) guideline emphasizes individualized selection of coronary revascularization strategy according to anatomy, clinical characteristics, expected completeness of revascularization, Heart Team assessment, and patient preferences [6,7]. Therefore, MICS-CABG should not be regarded as universally preferable to conventional CABG. Rather, it should be viewed as an alternative surgical access strategy in appropriately selected patients and experienced centers [16].

MICS-CABG also provides a foundation for hybrid coronary revascularization (HCR), combining minimally invasive surgical revascularization with PCI. HCR is particularly attractive in patients with multivessel CAD in whom LITA-LAD grafting is favored but selected non-LAD lesions are considered suitable for PCI [17,20]. Contemporary reviews emphasize that HCR may provide a compromise between the durability of an arterial LAD graft and the less invasive nature of PCI for selected non-LAD lesions [17,20]. However, the current study did not evaluate HCR and no conclusions regarding hybrid treatment can be drawn from the dataset.

Clinical implications

The current findings have several implications for minimally invasive cardiac-surgery programs. First, MICS-CABG can be used for substantial multivessel coronary revascularization, not merely isolated LAD disease. Second, three-graft procedures represented the predominant operative category. Third, early postoperative ventilation was relatively short, with a median duration of six hours. Fourth, the study identifies several important gaps in institutional data collection.

A standardized prospective MICS registry should include the following preoperative variables: Body mass index, left ventricular ejection fraction, Synergy Between Percutaneous Coronary Intervention With TAXUS and Cardiac Surgery (SYNTAX) score, left main coronary artery disease, number of diseased coronary vessels, previous percutaneous coronary intervention, previous myocardial infarction, peripheral vascular disease, chronic obstructive pulmonary disease, chronic kidney disease, and European System for Cardiac Operative Risk Evaluation II (EuroSCORE II).

Operative variables should include exact surgical approach, on-pump/off-pump status, CPB time, cross-clamp time, number of distal anastomoses, LITA use, radial artery use, saphenous vein use, total arterial revascularization, conversion to sternotomy, conversion to CPB, emergency conversion, and graft-flow assessment.

Postoperative outcomes should include operative mortality, 30-day mortality, stroke, myocardial infarction, atrial fibrillation, respiratory complications, acute kidney injury, reoperation for bleeding, transfusion, ICU stay, hospital stay, wound complications, and readmission. Long-term outcomes should include graft patency, repeat revascularization, major adverse cardiac and cerebrovascular events (MACCE), survival, recurrent angina, and quality of life.

Strengths

The present study has several strengths.

First, it represents a real-world institutional experience rather than a highly selected clinical trial population. Second, the cohort includes 149 minimally invasive coronary revascularization procedures, providing a meaningful initial institutional experience. Third, multivessel procedures predominated, with two-, three-, and four-graft operations accounting for the vast majority of identifiable graft procedures. Fourth, demographic and cardiovascular risk-factor data were available for essentially the entire cohort. Fifth, CPB, cross-clamp, ventilation, and chest drainage data were available in most patients.

Sixth, the analysis deliberately avoids interpreting missing complication fields as negative outcomes, reducing the risk of falsely favorable reporting.

Limitations

Retrospective Design

The retrospective design limits control over data quality, definitions, and completeness.

Single-Center Experience

The findings may not be generalizable to other institutions with different patient populations, surgical techniques, or levels of MICS experience.

No Conventional CABG Control Group

Without a contemporaneous sternotomy CABG group, the present study cannot determine whether MICS-CABG provides superior or equivalent outcomes.

Incomplete Complication Data

Outcome ascertainment was not uniform across all postoperative variables. Major predefined perioperative outcomes, including operative mortality, stroke, conversion to sternotomy, reoperation for bleeding, and new-onset atrial fibrillation, were systematically captured for the study cohort; however, documentation of other postoperative complications was incomplete and heterogeneous. Consequently, the study can report the specified major outcomes but cannot provide a reliable comprehensive overall complication rate. Consider this limitation when interpreting the overall perioperative safety profile of the procedure.

Incomplete Transfusion Data

Blood-product documentation was heterogeneous and did not consistently distinguish the type and number of blood products.

Lack of Anatomical Coronary Data

Although graft number was available, the dataset did not provide a standardized anatomical definition of multivessel CAD or SYNTAX score.

Lack of Complete Clinical Endpoints

Mortality, myocardial infarction, stroke, acute kidney injury, conversion to sternotomy, reoperation, ICU stay, hospital stay, and readmission were not sufficiently complete for analysis.

Heterogeneity of the Operative Strategy

The cohort included both on-pump and off-pump minimally invasive coronary revascularization procedures, resulting in some procedural heterogeneity. Combined CABG-valve procedures were excluded from the final study cohort.

Lack of Long-Term Follow-Up

Graft patency, survival, recurrent angina, repeat revascularization, and MACCE could not be evaluated.

## Conclusions

MICS-CABG may provide a feasible approach for multivessel coronary revascularization in appropriately selected patients. The predominance of multigraft procedures in this series indicates that a minimally invasive approach can be extended beyond isolated single-vessel revascularization in an experienced center.

However, these results represent a descriptive institutional experience and should not be interpreted as evidence of superiority over conventional CABG. Prospective studies with standardized reporting of major clinical outcomes, graft patency, repeat revascularization, and long-term survival are needed to further evaluate the safety, durability, and clinical effectiveness of MICS-CABG.
